# First Molecular Evidence and Phylogeny of *Hepatozoon* sp. and *Theileria* sp. in Saudi Rodents

**DOI:** 10.3390/vetsci12070608

**Published:** 2025-06-21

**Authors:** Sarra Farjallah, Abdulaziz Nasser Alagaili, Bandar H. AlOsaimi, Paolo Merella, Osama B. Mohammed, Nabil Amor

**Affiliations:** 1 Laboratory of Ecology, Biology and Physiology of Aquatic Organisms LR18ES41, Faculty of Sciences of Tunis, University of Tunis El Manar, Tunis 2092, Tunisia; 2 Department of Zoology, King Saud University, P.O. Box 2455, Riyadh 11451, Saudi Arabia; 3 Department of Veterinary Medicine, University of Sassari, Via Vienna, 2, 07100 Sassari, Italy

**Keywords:** *Hepatozoon*, *Theileria*, Saudi Arabia, rodents, *16S* rRNA gene, *18S* rRNA gene, haplotype analysis, phylogenetic analyses

## Abstract

Rodents are among the most abundant and ecologically diverse mammals, playing key roles in terrestrial ecosystems and often serving as reservoirs for various zoonotic and wildlife pathogens. Among these are protozoan parasites of the genera *Hepatozoon* and *Theileria*, which are known to infect a wide range of domestic and wild animals worldwide. However, little is known about the diversity and phylogenetic relationships of these hemoprotozoans in rodent hosts, particularly in the Arabian Peninsula. The aim of this study was to investigate the presence and genetic diversity of *Hepatozoon* sp. and *Theileria* sp. in rodents from different regions of Saudi Arabia and to determine potential reservoir species. A total of 111 rodents were captured and identified by molecular analysis of the mitochondrial *16S* rRNA gene. Screening for parasites was performed using PCR amplification of the *18S* rRNA gene, followed by sequencing, haplotype analysis, and phylogenetic reconstruction using both maximum likelihood and Bayesian inference methods. Our results represent the first molecular detection of *Hepatozoon* sp. in *Arvicanthis niloticus* (31.3%), *Gerbillus cheesmani* (26.5%), *G. nanus* (28.5%), and *Rattus rattus* (32.0%) and of *Theileria* sp. in *G. nanus* (21.5%) and *R. rattus* (24.0%) in Saudi Arabia. Haplotype network analysis revealed seven distinct *Hepatozoon* haplotypes forming a star-like cluster, suggesting host specificity. One divergent haplotype (Hap_2), 19 mutation steps apart, may represent a novel lineage. Phylogenetic analyses grouped Saudi *Hepatozoon* sequences with those from reptiles and rodents, forming a clade distinct from sequences isolated from felids and canids. In contrast, *Theileria* sequences showed low diversity, clustering with a single widespread haplotype found in rodents and ruminants in several regions. These findings significantly expand the current knowledge on rodent-associated apicomplexan parasites in Saudi Arabia, revealing novel *Hepatozoon* haplotypes and highlighting the role of rodents in the transmission of reptile-associated *Hepatozoon* spp. This study provides basic molecular data crucial to understanding host–parasite relationships and the potential public and veterinary health implications of these parasites in arid ecosystems.

## Simple Summary

Apicomplexan species of the genera *Hepatozoon* and *Theileria* are known to infect both domestic and wild animals. This study, performed using molecular tools, provides the first report of *Hepatozoon* sp. in rodent species (*Arvicanthis niloticus*, *Gerbillus cheesmani*, *G. nanus*, and *Rattus rattus*) from Saudi Arabia. Additionally, *Theileria* sp. was detected in *G. nanus* and *R. rattus*. Genetic analyses revealed that Saudi *Hepatozoon* haplotypes form a star-like haplogroup, indicating host specificity to local rodent species. Phylogenetic analyses generated trees with similar topologies, revealing two sister clades: one comprising *Hepatozoon* spp. sequences from rodents and reptiles forming a distinct clade, separate from those found in felids and canids. These findings confirm that rodents play a key role in the epidemiology of reptile-associated *Hepatozoon* spp. rather than species linked to Carnivora. The *Hepatozoon* sequences from Saudi Arabia formed a well-supported cluster, revealing novel haplotypes primarily associated with rodents. This study also highlights the lack of genetic diversity in *Theileria* sp. in Saudi Arabia, with a single haplotype clustering with sequences from ruminants and rodents across multiple regions.

## 1. Introduction

Tick-borne diseases are among the most significant diseases impacting both human and veterinary health worldwide, second only to mosquito-borne diseases. Among them, hepatozoonosis is a vector-borne infectious disease that has been increasingly studied in the past decade due to its veterinary importance [1,2]. Hepatozoonosis is caused by apicomplexan protozoans such as *Hepatozoon* spp., which have been described in both domestic and wild mammals. *Hepatozoon* spp. are intracellular hemogregarine parasites with a heteroxenous life cycle, in which ticks of the genera *Rhipicephalus* and *Ixodes* serve as definitive hosts, while vertebrates act as intermediate or paratenic hosts. The primary mode of transmission to vertebrate hosts is through the ingestion of infected ticks. Additional transmission routes include transplacental transfer of sporozoites from female ticks to their eggs [3], ingestion of tissues from paratenic hosts harboring sporozoites, and predation on vertebrates infested with infected ticks [4,5]. Other apicomplexan parasites, such as *Theileria* spp., are intracellular protozoa of the family Theileridae. They infect lymphocytes, causing various clinical signs of theileriosis in wild and domestic ruminants [6]. Transmission to vertebrate hosts occurs through the salivary glands of infected ticks belonging to the genera *Amblyomma*, *Haemaphysalis*, *Hyalomma*, *Ixodes*, and *Rhipicephalus* [3].

Protozoans of the genera *Hepatozoon* and *Theileria* have been linked to infections in domestic mammals in Saudi Arabia [7,8]. A recent study reported, for the first time, the presence of *H. canis* in camels from Riyadh Province, Saudi Arabia [9]. This occurrence may be attributed to the broad host range and widespread distribution of *H. canis* [3] and its vectors, particularly *Rhipicephalus sanguineus* sensu lato, which is known to infect dogs in this region [10]. *Theileria* spp. have been reported in small ruminants, with *T. ovis* found in sheep and goats in Jeddah, Saudi Arabia [8], while *T. annulata* is found in cattle [7].

Most emerging zoonotic pathogens originate from wild animals [4,5]. Wild fauna has always been considered a key factor in the emergence and re-emergence of zoonotic diseases in nature [11]. Several pathogenic *Hepatozoon* spp. have been identified in these hosts, including *H. pyramidumi*, *H. bashtari*, and *H. aegypti*, which have been described from the blood of the Egyptian saw-scaled viper (*Echis pyramidum*), the painted saw-scaled viper (*E. coloratus*), and the diadem snake (*Spalerosophis diadema*), respectively, in Saudi Arabia [12,13]. Recently, Mohammed et al. [14] reported the detection of *Theileria* spp. in desert hedgehogs (*Paraechinus aethiopicus*) in Saudi Arabia, marking the first documented occurrence of this pathogen in this species within the region.

Nevertheless, there is a lack of information regarding *Hepatozoon* and *Theileria* infections in wild animals from Saudi Arabia, particularly in rodents. Despite the rich rodent fauna, these animals have not been systematically studied for the presence of these parasites, leaving significant gaps in the understanding of their epidemiology and genetic diversity in the region. Wild rodents are recognized as reservoirs for various zoonotic pathogens and can transmit diseases to humans [15]. Rodents exhibit high adaptability across diverse habitats worldwide. They play a critical role in the life cycle of several tick species, serving as hosts at different developmental stages. Their omnivorous diet, which includes small vertebrates and invertebrates, likely facilitates the cross-species transmission of *Hepatozoon* spp. and *Theileria* spp. [14,15]. Research on small mammals remains limited in this area, suggesting that the diversity of these protozoans may be significantly underrepresented, particularly among rodents. Substantial knowledge gaps remain regarding the identification of reservoir species and their geographic distribution [4,14]. Although reservoirs are widespread, they are not accurately identified, and most studies rely on small sample sizes from limited geographic areas.

Conventional methods, such as optical microscopy, are used to identify parasitic species based on their morphological characteristics, life history, host symptoms, infected vertebrate taxa, and arthropod vectors [16]. However, the morphological characteristics of blood parasites can vary depending on the infection, especially when parasites are sampled from different hosts [17]. The development of more sensitive molecular tools, such as PCR and sequencing, has enabled the accurate identification of apicomplexan protozoans. Several techniques have been described, with conventional PCR on blood samples proving more sensitive and specific than classical methods for diagnosing hemoparasite species [18,19]. Currently, molecular approaches relying on *18S* rRNA gene sequence variations have proven to be highly valuable complementary tools for distinguishing closely related *Hepatozoon* and *Theileria* species [8,20,21,22,23,24,25].

Considering the limitations of morphological taxonomy, accurate host species identification is crucial for establishing a precise correlation between host species and potential pathogen prevalence. The mitochondrial *16S* ribosomal RNA (*16S* rRNA) gene, known for its high variability, is widely used in molecular taxonomy as a reliable marker for species identification. Its sequencing has proven particularly valuable for distinguishing closely related rodent species [26]. Due to its effectiveness, the *16S* rRNA gene is commonly employed in phylogenetic and taxonomic studies [26,27].

Moreover, the identification of potential new pathogens, often resulting from the spread of new wild reservoir host species, underscores the importance of enhanced surveillance and thorough molecular characterization of potential reservoir hosts [21,22,28,29]. In this context, the aims of the current study were to detect *Hepatozoon* sp. and *Theileria* sp. and to characterize their rodent host species across different regions of Saudi Arabia using molecular tools, contributing to a deeper understanding of the epidemiology of these pathogens and their impact on wildlife, domestic animals, and public health.

## 2. Materials and Methods

### 2.1. Sample Collection

Between 2021 and 2023, blood samples from different species of rodents (111 specimens) were obtained from twelve localities of Saudi Arabia (Table 1). Rodent species were identified morphologically using a taxonomic guide, focusing on key morphological traits, such as size, fur color, and tail length, which serve as distinguishing characteristics for differentiating rodent species [30]. Sampled rodents included five species: *Arvicanthis niloticus* (African grass rat, *n* = 32), *Gerbillus cheesmani* (Cheesman’s gerbil, *n* = 34), *G. dasyurus* (Wagner’s gerbil, *n* = 6), *G. nanus* (Baluchistan gerbil, *n* = 14), and *Rattus rattus* (black rat, *n* = 25) (Table 1). Rodents were captured using Sherman traps strategically placed in tunnels identified as active through visual assessments of rodent activity. At each site, the traps were checked daily for one week. Field method guidelines from Herbreteau et al. [31] were adhered to during the capture process, ensuring minimal stress for the animals. A minimally invasive technique, tail vein puncture, was employed to obtain blood samples, providing sufficient material for laboratory analysis, as described by Alotaibi et al. [32]. After sampling, the rodents were returned to their original capture sites to minimize disturbance to the local populations. All procedures complied with animal welfare standards to ensure ethical treatment (Ethics Reference No: KSU-SE-21-07). The collected samples were stored frozen at −20 °C until further molecular analysis.

### 2.2. Host Species Molecular Characterization

Genomic DNA was extracted from rodent whole-blood samples (30–50 μL) collected in EDTA. Initially, erythrocytes were lysed using ammonium chloride potassium (ACK) buffer to isolate white blood cells (WBCs). The WBCs were then resuspended in PBS, lysed using a lysis buffer containing sodium dodecyl sulfate (SDS), and digested with proteinase K at 55 °C for 30 min to ensure efficient cell and nuclear lysis and protein degradation. The mixture was centrifuged at 13,000 rpm for 10 min to pellet the proteins. DNA in the supernatant was then precipitated with isopropanol. The resulting DNA pellet was washed with 70% ethanol, air-dried, and resuspended in Tris-EDTA (TE) buffer. The extracted DNA was stored at −20 °C and was suitable for PCR amplification, enabling both host species identification and the detection of protozoan infections [33]. A partial fragment of the 16S rRNA gene from the 111 rodent specimens was amplified using the primer pair 16sa-L (5′-CGCCTGTTTATCAAAAACAT-3′) and 16sb-H (5′-CCGGTCTGAACTCAGATCACGT-3′) [34]. The thermal cycling conditions for amplification included an initial denaturation at 94 °C for 3 min, followed by 35 cycles of denaturation at 94 °C for 30 s, annealing at 50 °C for 30 s, and elongation at 72 °C for 1 min, with a final extension at 72 °C for 10 min. The obtained sequences were edited and aligned using Unipro UGENE 1.3 [35]. Multiple sequence alignments were performed using the ClustalW v2.1 program integrated into UGENE, with reference sequences for the 16S gene obtained from GenBank via the BLAST 1.4.0 algorithm [36]. The optimal evolutionary model and partitioning scheme for the 16S gene were determined using PartitionFinder 2.1 [37], identifying GTR + G as the best substitution model for the dataset under maximum likelihood (ML). A maximum likelihood (ML) phylogenetic tree was constructed using RAxML [38], with bootstrap support assessed through 2000 pseudoreplicates.

### 2.3. Molecular Identification of Hepatozoon and Theileria

#### 2.3.1. DNA Extraction and *18S* rRNA Gene Amplification and Sequencing

PCR reactions targeted a fragment of the *18S* rRNA gene to detect *Hepatozoon* sp. and *Theileria* sp. The PCR was performed using a set of primers: RLB-F2 (5′-GAC ACA GGG AGG TAG TGA CAA G-3′) and RLB-R2 (5′-CTA AGA ATT TCA CCT CTG ACA GT-3′), amplifying a fragment of 460–540 bp from the *18S* rRNA gene spanning the V4 region, with both negative extraction and negative PCR controls [39]. In a 25 μL reaction volume, 1× PCR buffer, 2.5 mM MgCl_2_, 0.2 mM of each dNTP, 0.5 μM of each primer, 1 U of Taq DNA polymerase (Bioline, Memphis, TN, USA), ddH_2_O, and approximately 20 ng of template DNA were used for the PCR amplification. The PCR conditions included an initial step of 3 min at 37 °C, followed by 10 min at 94°C, then 10 cycles of 94 °C for 20 s, 67 °C for 30 s, and 72 °C for 30 s, with the annealing temperature decreasing by 1 °C every second cycle (touchdown PCR). The reaction was then followed by 40 cycles of denaturation at 94 °C for 20 s, annealing at 57 °C for 30 s, and extension at 72 °C for 30 s, with a final extension at 72 °C for 10 min [40]. PCR products were separated by gel electrophoresis (1% agarose) with the molecular weight marker HyperLadder 100 bp (Bioline Reagents Ltd., London, UK). The PCR products were sequenced at the Macrogen sequencing facility (Macrogen Inc., Seoul, Korea).

#### 2.3.2. Haplotype and Phylogenetic Analysis of *Hepatozoon* and *Theileria*

The obtained sequences were manually examined and aligned using ClustalW software, integrated into Mega X version 10.2.5 [41]. Sequence alignments included comparative sequences of species parasitizing dogs, cats, rodents, reptiles, and ticks, obtained from GenBank using the BLAST algorithm (http://www.ncbi.nlm.nih.gov/BLAST, accessed 2 February 2025) and aligned with those generated in this study. Genetic diversity among *Hepatozoon* sequences detected in the sampled rodents was assessed using DnaSP v.5.10.01 [42] based on *18S* rRNA analysis. The software estimated haplotype (H), haplotype (Hd), and nucleotide (Pi) diversities; the average nucleotide differences between sequences (k); and the number of polymorphic sites and insertions/deletions (S). Haplotype networks were constructed using PopArt [41] to illustrate the evolutionary relationships between haplotypes detected in this study and those reported from different geographic regions. The median-joining (MJ) network algorithm was used with default parameters (equal character weight 10, transition/transversion weight 1:1, and connection cost as the criterion). The optimal evolutionary model was determined using PartitionFinder 2.1 [37]. The GTR + G substitution model was applied for both maximum likelihood (ML) and Bayesian analyses of the *18S* dataset. Phylogenetic trees for the *18S* dataset were constructed using ML and Bayesian (BI) analyses with Mega X version 10.2.5 and MrBayes version 3.2.6 [43], respectively. The phylogenetic tree was rooted using sequences from *Babesia equi* (from *Equus caballus*, LC008132) as an outgroup.

## 3. Results

### 3.1. Characterization of Rodent Species

**Figure 1:**
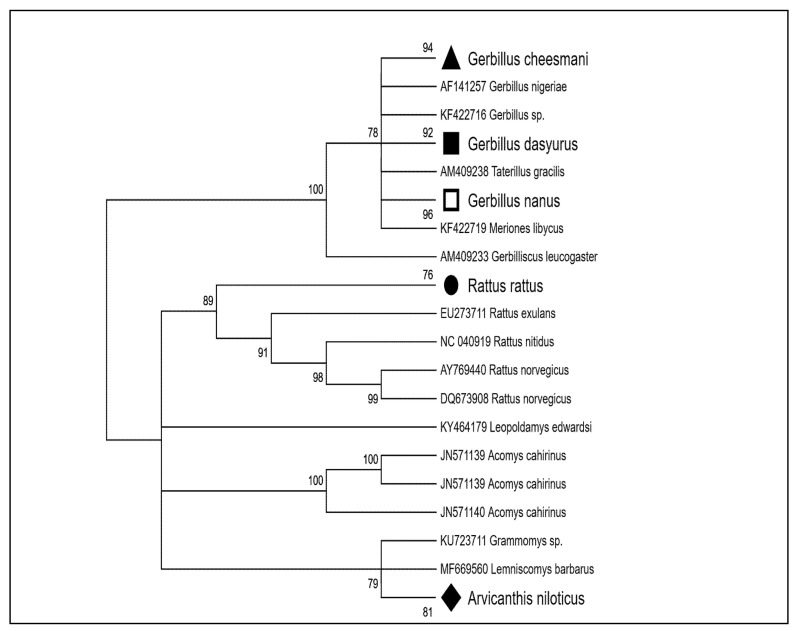
Maximum likelihood phylogenetic tree of rodent species based on *16S* sequences (750 bp). Different taxonomic branches are represented by the following shapes: ▲: *Gerbillus cheesmani*, ■: *G. dasyurus*, □: *G. nanus*, ●: *Rattus rattus*, ♦: *Arvicanthis niloticus*.

Among the 111 specimens of rodents sampled, 750 bp sequences of the *16S* gene were successfully obtained. Analysis of the gene translation confirmed the absence of pseudogenes, with nucleotide variation mainly occurring at the third codon position. Alignment of the *16S* sequences revealed 197 polymorphic sites and 18 haplotypes, defining five distinct rodent species. Using the *16S* sequences, along with reference sequences from databases, a maximum likelihood (ML) phylogenetic tree was constructed to assess the relationships within and between species (Figure 1). The haplotypes were divided into two major clades corresponding to the Murinae and Gerbillinae superfamilies, with five highly supported subclades (>76% bootstrap support). These subclades corroborated the morphological identification of the species (Table 1).

### 3.2. Molecular Characterization of Hepatozoon sp. and Theileria sp.

#### 3.2.1. Prevalence of *Hepatozoon* and *Theileria* in Examined Rodents

Based on the analysis of *18S* gene sequences, 9 and 31 rodent samples tested positive for *Theileria* sp. and *Hepatozoon* sp. infections, with a total prevalence 8% and 28%, respectively. Table 1 shows the prevalence of *Theileria* sp. and *Hepatozoon* sp. in the different host species and localities. The prevalence of *Hepatozoon* sp. in different host species, regardless of locality, ranged from 26.5% in *G. cheesmani* to 32% in *R. rattus*. For *Theileria* sp., prevalence ranged from 21.5% in *G. nanus* to 24% in *R. rattus*. Only *G. nanus* and *R. rattus* harbored both pathogens. No infections were observed in *G. dasyurus* (Table 1). Concerning localities, regardless of host species, only in Baljurashi *R. rattus* was infected with both pathogens, with a prevalence of 40% for *Hepatozoon* sp. and 60% for *Theileria* sp. Neither *Hepatozoon* sp. nor *Theileria* sp. was detected in rodents from seven and ten of the twelve localities studied, respectively (Table 1).

#### 3.2.2. Population Genetic Analysis

**Figure 2:**
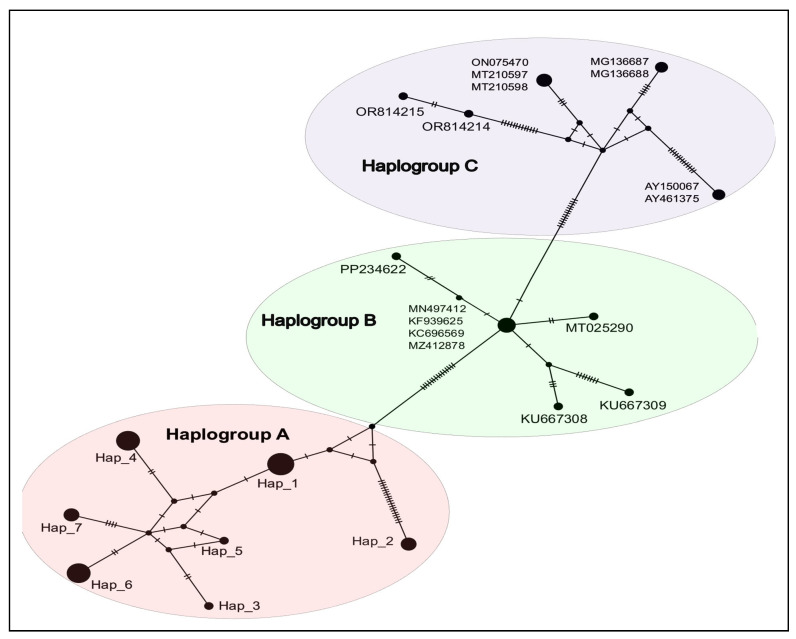
Haplotype network for *Hepatozoon* spp. based on *18S* rRNA sequences obtained in this study (Haplogroup A, Hap1-7) and those available in the GenBank database. The hatch marks represent the number of mutational steps between haplotypes. The black circles indicate alternative unsampled haplotypes. Abbreviations: H1–H7, haplotype IDs (see Table 2).

The multiple alignments of the partial *18S* rRNA gene (442 bp) included sequences from *Hepatozoon* spp. (*n* = 31) and *Theileria* spp. (*n* = 9) obtained from various host species, localities, and reference sequences available in GenBank. The alignment of *Hepatozoon* sp. *18S* sequences revealed 85 polymorphic sites and 76 parsimony-informative sites. These haplotypes were submitted to GenBank under accession numbers PV342388–PV342394 (Table 2). The *Hepatozoon 18S* sequences showed high haplotype diversity (Hd = 0.916 ± 0.019 S.D.) but low nucleotide diversity (Pi = 0.04458 ± 0.00491 S.D.), with an average number of nucleotide differences (k) of 18.859. In Saudi Arabia, *Hepatozoon* sp. was distributed across seven haplotypes (Hap_1–Hap_7), with no haplotypes shared across the full geographic range of the host species (Table 1 and Table 2). The major haplotype, Hap_1 (*n* = 9), was found exclusively in *G. cheesmani* from Baljurashi. Hap_2 and Hap_3 were detected only in *G. nanus* from Ahad Almasarha (*n* = 3) and Al Radha (*n* = 1), respectively. Hap_4 and Hap_5 were identified in *R. rattus*, with Hap_4 occurring in Samtah (*n* = 2), Al Dair (*n* = 1), and Baljurashi (*n* = 4), while Hap_5 was restricted to Al Aridhah (*n* = 1). The rodent *A. niloticus* carried two haplotypes, Hap_6 (*n* = 7) and Hap_7 (*n* = 3), both exclusive to Baljurashi. The Saudi Arabian haplotypes formed a single haplogroup (haplogroup A) with a star-like structure, separated from the referenced sequences by 15 mutation steps (Figure 2). Hap_1 and Hap_3 to Hap_7 were separated by 1 to 4 mutation steps, while Hap_2, found only in *G. nanus* from Ahad Almasarha, was 19 mutation steps apart from the other Saudi Arabian haplotypes (Figure 2). In contrast, the *Theileria* sp. *18S* sequences were highly conserved, with a single haplotype identified in *G. nanus* from Al Darb (*n* = 3) and *R. rattus* from Baljurashi (*n* = 6). This haplotype was submitted to GenBank under accession number PV342395.

#### 3.2.3. Phylogenetic Analysis

**Figure 3:**
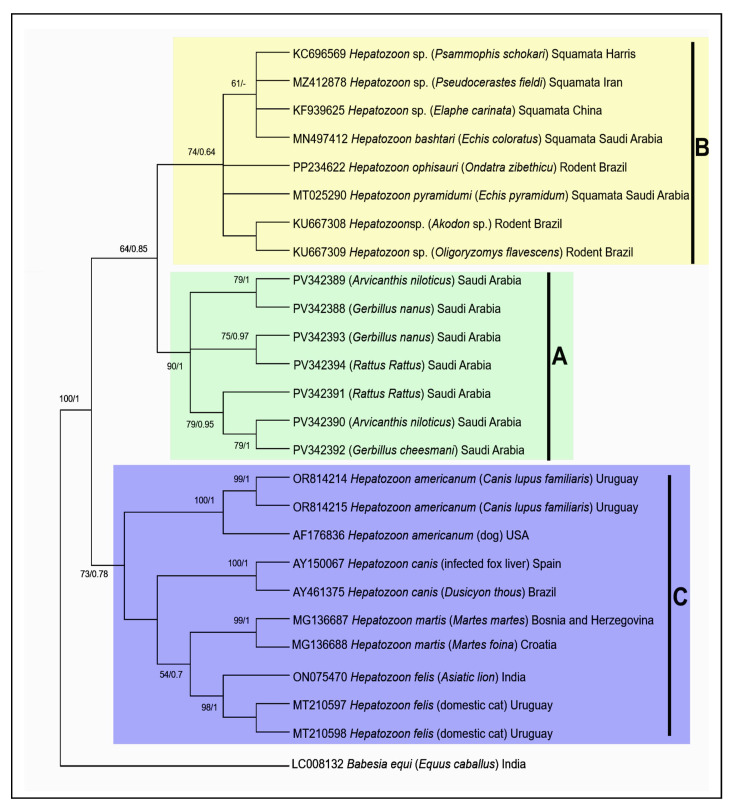
Maximum likelihood and Bayesian phylogenetic tree of *Hepatozoon* spp. based on *18S* rRNA sequences obtained in this study and those available in the GenBank database. Support values for each node represent ML bootstrap values (right) and BI posterior probabilities (left). Only nodal support values > 50% are shown. Cluster A: Parasite sequences isolated from Saudi Arabian rodents, Cluster B: Reference sequences of parasites infecting rodents and reptiles, Cluster C: Reference sequences of parasites infecting felid and canid hosts.

The ML and BI analyses generated trees with similar topologies, with *Hepatozoon* spp. sequences clustering into two highly supported sister clades (BS/PI: 100/1) (Figure 3). The first clade was subdivided into cluster A, containing sequences from parasites isolated from various rodent species in Saudi Arabia in the present study, and cluster B, which included GenBank sequences from parasites infecting rodents and reptiles. The second clade (cluster C) comprised previously published sequences from felid and canid hosts across several geographical regions (Brazil, Bosnia, Croatia, India, Uruguay, Spain, USA). In the first clade, *Hepatozoon* spp. sequences from *Psammophis schokari* (KC696569), *Pseudocerastes fieldi* (MZ412878), and *Elaphe carinata* (KF939625) clustered with previously published sequences of *H. bashtari* (MN497412) from *E. coloratus* and *H. pyramidum* (MT025290) from *E. pyramidum* in Saudi Arabia. This reptile-associated *Hepatozoon* group also clustered with sequences of *Hepatozoon* spp. (KU667308; KU667309) and *H. ophisauri* (PP234622) from rodent species (*Akodon* sp., *Oligoryzomys flavescens*, *Ondatra zibethicus*), supported by a bootstrap value of 74 and PI of 0.64. Additionally, *Hepatozoon* sequences from Saudi Arabian rodent species (*A. niloticus*, *G. cheesmani*, *G. nanus*, *R. rattus*) formed a well-supported cluster (BS/PI: 90/1), reinforcing the reliability of this grouping. *Hepatozoon martis* (MG136687; MG136688) from mustelids (*Martes martes*, *M. foina*) and *H. felis* (ON075470; MT210597; MT210598) from both domestic and wild cats (including the Asiatic lion and domestic cat) formed a distinct, well-supported cluster. This feline-associated cluster was closely related to *H. canis* (AY150067; AY461375) sequences from fox species (*Dusicyon thous*) in Spain and Brazil, as well as *H. americanum* (OR814214; OR814215; AF176836) sequences from dogs (*C. lupus familiaris*) in Uruguay, Spain, and the USA. High bootstrap and PI values (73/0.78) further supported the reliability of these groupings.

**Figure 4:**
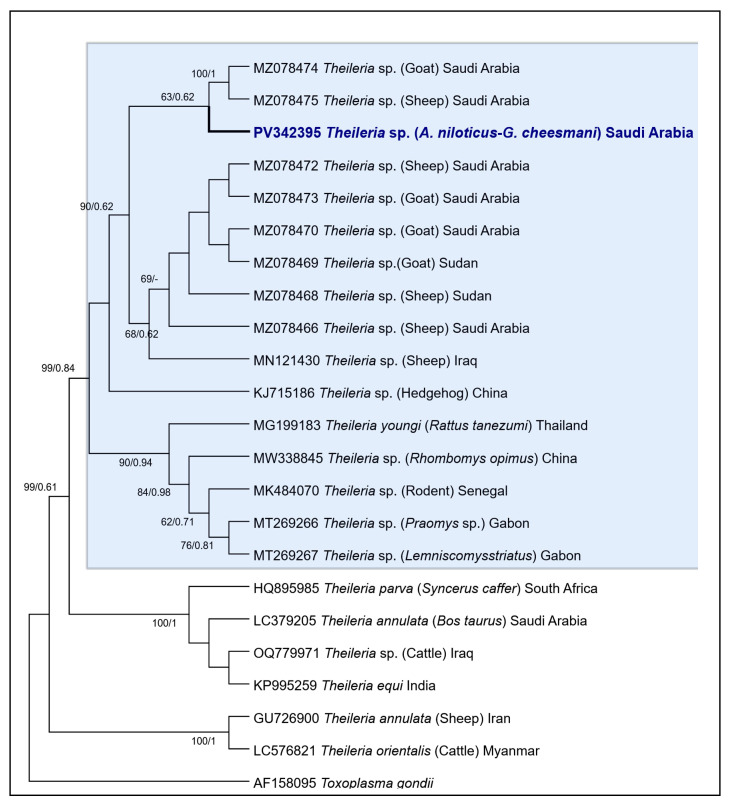
Maximum likelihood and Bayesian phylogenetic tree of *Theileria* spp. based on *18S* rRNA sequences obtained in this study and those available in the GenBank database. Support values for each node represent ML bootstrap values (right) and BI posterior probabilities (left). Only nodal support values > 50% are shown. The bold branch represents the *Theileria* haplotype from Saudi Arabia.

The phylogenetic trees constructed using ML and BI yielded similar topologies for *Theileria* spp. The *18S* sequences from Saudi Arabia (PV342395), isolated from *G. nanus* and *R. rattus* in this study, clustered with previously published sequences from Saudi Arabia (MZ078466, MZ078470, MZ078472-MZ078475), Sudan (MZ078468, MZ078469), and Iraq (MN121430), all isolated from sheep and goats, forming a highly supported group (BS/PI: 90/0.62) (Figure 4). The phylogenetic trees constructed using ML and BI methods yielded similar topologies for *Theileria* spp. The *Theileria 18S* sequences from Saudi Arabia (PV342395), isolated from *G. nanus* and *R. rattus* in this study, clustered with previously published sequences from Saudi Arabia (MZ078466, MZ078470, MZ078472–MZ078475), Sudan (MZ078468, MZ078469), and Iraq (MN121430), all isolated from sheep and goats, forming a well-supported clade (BS/PI: 90/0.62) (Figure 4). Additionally, sequences of *Theileria* spp. from China (KJ715186, MW338845), Gabon (MT269266, MT269267), and Senegal (MK484070) from various rodent species (*Hedgehog*, *Rhombomys opimus*, *Praomys* sp., *Lemniscomys striatus*) were also grouped in the same genotype cluster. The *Theileria youngi* sequence (MG199183) from Thailand, isolated from *Rattus tanezumi*, clustered in the same cluster. High bootstrap and PI values (99/0.84) further supported the reliability of these groupings (Figure 4).

## 4. Discussion

Previous studies have reported the presence of different apicomplexan parasites in rodents from Saudi Arabia, including *Babesia* [7], *Cryptosporidium* [53,54], *Haemogregarina* [13,55], *Neospora* [56], *Sarcocystis* [57,58], and *Toxoplasma* [59]. Several *Hepatozoon* species have also been documented in Saudi Arabia, including *H. canis* in mammals such as camels and dogs, *H. hemprichii* in the reptile *Scincus hemprichii*, *H. pyramidumi* sp. n. from *E. pyramidum*, and *H. bashtari* in *E. coloratus*, as well as in tick species such as *H. ayorgbor* in *R. haemaphysaloides* and *H. colubri* in *H. sulcata* and *Hyalomma anatolicum*. Additionally, *Theileria* spp. have been reported in hedgehogs (*P. aethiopicus*) and *T. ovis* in sheep, goats, and cattle [7,8,14]. Although *Hepatozoon* spp. and *Theileria* spp. have been confirmed in mammals and reptiles, studies on their occurrence in rodents remain limited. In this study, *16S* gene analysis of 111 sampled rodents identified five species from the superfamilies Murinae and Gerbillinae, each characterized by distinct habitat preferences. This molecular approach has proven essential in addressing significant gaps in identifying apicomplexan reservoirs and their geographic distribution. The diversity of rodent hosts, combined with the variety of their ecological niches, may play a key role in shaping the distribution and transmission dynamics of apicomplexan parasites. The habitat-specific occurrence of Murinae in farms and mountainous regions and of Gerbillinae in desert and coastal sand dunes aligns with previous research on their morphology, distribution, and ecological adaptations [30,32,60]. These findings highlight the need for further investigations into the potential role of different rodent species as reservoirs for *Hepatozoon* and *Theileria* in Saudi Arabia.

Based on *18S* gene sequence analysis, this study provides the first report of *Hepatozoon* sp. and *Theileria* sp. in rodents from Saudi Arabia, with a total prevalence of 28% and 8%, respectively. This is the first description of *Hepatozoon* sp. in *G. cheesmani* (26.5%), *G. nanus* (28.5%), *A. niloticus* (31.25%), and *R. rattus* (32%), as well as of *Theileria* sp. in *G. nanus* (21.5%) and *R. rattus* (24%) in this region. Compared to these findings, Chandra et al. [10] reported a low prevalence of *H. canis* in dogs (5.7%), while Alanazi et al. [61] observed an even lower prevalence (0.6%) in *Camelus dromedarius* in Riyadh, Saudi Arabia. However, the prevalence of *Theileria* sp. (8%) in rodents in the present study was lower than the previously reported values in hedgehogs (45.5%), sheep (57.8%), and goats (51.9%) in Saudi Arabia. Variations in the prevalence of apicomplexan parasites can be attributed to several factors, including the geographic distribution, the abundance of vector populations [3], climatic conditions, host immune status, and the specific populations targeted [62,63,64,65].

Phylogenetic analysis showed clustering patterns of *Hepatozoon* spp. consistent with those reported by Santos et al. [66]. Based on *18S* rRNA gene sequences, *Hepatozoon* spp. from rodents and reptiles in Saudi Arabia and other regions formed a distinct clade (A + B), clearly separate from the clade C, which comprised sequences from felids and canids across various geographical regions. This finding supports previous studies indicating that *Hepatozoon* spp. from rodents are closely related to those from reptiles but are phylogenetically distant from species infecting canids and felids. Rodent-associated *Hepatozoon* species thus appear to be excluded from the epidemiological cycles of *Hepatozoon* infecting domestic and wild felids and canids, as previously reported [67,68,69,70,71]. Within this clade, the subclade B included *Hepatozoon* sequences from reptiles, such as *Psammophis schokarii*, *P. fieldi*, *E. carinata*, *E. coloratus*, and *E. pyramidum*. This group clustered with *Hepatozoon* sp. and *H. ophisauri* from rodent species, including *Akodon* sp., *O. flavescens*, and *O. zibethicus*. Notably, *Hepatozoon* sequences from Saudi Arabian rodents (*A. niloticus*, *G. nanus*, *G. cheesmani*, *R. rattus*) formed a well-supported subclade (A), further reinforcing the robustness of this phylogenetic structure. The present findings suggest that rodents from Saudi Arabia may act as paratenic or even intermediate hosts for *Hepatozoon* infections in reptiles, although further investigation is needed to confirm this hypothesis.

This study provides the first assessment of the nucleotide diversity and genetic structure of *Hepatozoon* spp. in rodent samples from Saudi Arabia, based on the *18S* rRNA gene, and identified seven haplotypes (Hap_1–Hap_7). The haplotype diversity of *Hepatozoon* spp. in rodents (Hd = 0.916) was higher than that reported in Brazilian rodents (Hd = 0.426) by Perles et al. [71] and comparable to the diversity observed in Chilean rodents (Hd = 0.933) by Alabí et al. [20]. A distinct haplogroup (C) was exclusively associated with *Hepatozoon* from canids, while haplogroup B comprised sequences from rodents and reptiles retrieved from GenBank. Notably, the haplotypes identified in this study formed a separate haplogroup (A) with a star-like pattern, differing by fifteen mutational steps from previously reported sequences. This pattern suggests that these haplotypes may be specific to rodent species in Saudi Arabia. None of the *Hepatozoon 18S* rRNA sequences available in GenBank matched 100% with the haplotypes identified in Saudi rodents, indicating the presence of novel *Hepatozoon* haplotypes. These Saudi haplotypes appear to be predominantly associated with rodents and reptile-related *Hepatozoon* species. The possible role of rodents in the epidemiology of reptile-associated *Hepatozoon* spp. [71,72,73], rather than Carnivora-related species, is supported by the current findings and previous phylogenetic analyses. Indeed, the haplotype analysis network revealed a potential haplotype affinity to certain rodent species, regardless of geographic area. In Saudi Arabia, a variety of haplotypes were associated with single host species: *G. cheesmani* (Hap_1); *G. nanus* (Hap_2 and Hap_3); *R. rattus* (Hap_4 and Hap_5); and *A. niloticus* (Hap_6 and Hap_7). Interestingly, Hap_2, detected exclusively in *G. nanus*, differed by 19 mutation steps from the other Saudi Arabian haplotypes, suggesting the emergence of a new lineage specific to *G. nanus*. Haplotype diversity is influenced by multiple factors, including the life history of parasites and the evolutionary dynamics of hosts [74]. In general, *Hepatozoon* spp. are known to have low host specificity [75]. Different rodent groups and genera may harbor distinct *Hepatozoon* haplotypes. However, host preference for *Hepatozoon* haplotypes in rodents has been previously reported in Finland, Estonia, Russia, Poland, Nigeria, and Chile [73,76,77,78]. Therefore, the structure of rodent populations may influence the occurrence of certain *Hepatozoon* haplotypes. Further molecular characterization based on rapidly evolving genes is needed to confirm the hypothesis of a *Hepatozoon-G. nanus* lineage.

Regarding the genetic diversity and evolutionary relationships of *Theileria* spp. based on *18S* rRNA gene sequences, this study showed low genetic diversity among *Theileria* sp. in Saudi Arabia, with only a single haplotype identified in *G. nanus* and *R. rattus*. This haplotype closely clustered with sequences from ruminants in Saudi Arabia, Sudan, and Iraq, as well as with rodent-derived sequences from China, Gabon, and Senegal, suggesting a highly conserved genotype across diverse hosts and regions [8,15,79,80,81]. The occurrence of *Theileria* sp. in Saudi Arabia is not unexpected, given previous reports [7,8,14]. However, the observed similarity between the obtained sequences and *T. youngi* (MG199183) from Thailand, isolated from *R. tanezumi* [82], which was grouped in the same cluster, is a result that deserves to be further investigated in future studies.

## 5. Conclusions

In conclusion, this study provides the first molecular evidence of *Hepatozoon* sp. and *Theileria* sp. in rodents from Saudi Arabia, expanding the known host range in the region. It also contributes to the understanding of *Hepatozoon* spp. diversity by identifying novel haplotypes that appear to be unique to Saudi Arabian rodents and closely related to those previously reported in rodents and reptiles. These findings highlight the need for further research on the genetic diversity of *Hepatozoon* spp., including a broader sampling of rodent and reptile species and definitive hosts from Saudi Arabia, along with the analysis of additional molecular markers. Moreover, future studies should emphasize the conservation implications for wildlife, particularly in terms of veterinary conservation medicine and wildlife management, due to the potential impact of these species on animal health.

## Figures and Tables

**Table 1:** Presence of *Hepatozoon* spp. and *Theileria* spp. in rodent species from Saudi Arabia. N: number of individuals per rodent species, *n*: number of individuals per locality.

Rodent Species	Localities	Geographical Coordinates	*Hepatozoon* spp. PCR Positive %	*Theileria* spp. PCR Positive %
*A. niloticus* (N = 32)	Baljurashi (*n* = 32)	19°51′34″ N 41°33′26″ E	31.25%	-
*G. cheesmani* (N = 34)	Baljurashi (*n* = 34)	19°51′34″ N 41°33′26″ E	26.47%	-
*G. dasyurus* (N = 6)	Baljurashi (*n* = 6)	19°51′34″ N 41°33′26″ E	-	-
*G. nanus* (N = 14)			28.57%	21.43%
	Al Darb (*n* = 4)	17°44′00.2″ N 42°15′23.3″ E	-	75.00%
	Bish (*n* = 2)	17°34′41.4″ N 42°31′03.2″ E	-	-
	Ahad Al Msarha (*n* = 3)	16°42′34.7″ N 42°56′12.7″ E	100.00%	-
	Wadi Sabya (*n* = 2)	17°09′30.5″ N 42°54′45.1″ E	-	-
	Wadi Reem (*n* = 1)	23°57′52.2″ N 39°27′27.2″ E	-	-
	Al Radha (*n* = 2)	17°31′05.3″ N 42°24′40.2″ E	50.00%	-
*R. rattus* (N = 25)			32.00%	24.00%
	Samtah (*n* = 2)	16°35′29″ N 42°56′22″ E	100.00%	-
	Sabya (*n* = 3)	17°8′56.3″ N 42°37′33.3″ E	-	-
	Al Aridhah (*n* = 4)	17°01′36.8″ N 43°02′42.1″ E	25.00%	-
	Fifa (*n* = 2)	17°15′0″ N 43°06′0″ E	-	-
	Al Dair (*n* = 4)	17°20′43.4″ N 43°07′47.0″ E	25.00%	-
	Baljurashi (*n* = 10)	19°51′34″ N 41°33′26″ E	40.00%	60.00%

**Table 2:** Haplotype identification of *Hepatozoon* spp. from rodent species obtained in this study, along with published sequences used in the phylogenetic analysis, including their geographic locations and GenBank accession numbers. Hap_1–Hap_7: *Hepatozoon* haplotypes obtained in this study.

Species		Host/Geographic Origin	Geographic Origin	Accession Numbers	References
*Hepatozoon* sp.		*Akodon* sp.	Brazil	KU667308	[44]
		*Oligoryzomys flavescens*	Brazil	KU667309	
*Hepatozoon bashtari*		*Echis coloratus*	Saudi Arabia	MN497412	[13]
*Hepatozoon* sp.		*Elaphe carinata*	Saudi Arabia	KF939625	[45]
		*Psammophis schokari*	North Africa	KC696569	[46]
		*Pseudocerastes fieldi*	Iran	MZ412878	[47]
*Hepatozoon americanum*		*Canis lupus familiaris*	Uruguay	OR814214	[48]
		*Canis lupus familiaris*	Uruguay	OR814215	
*Hepatozoon ophisauri*		*Ondatra zibethicus*	USA	PP234622	[49]
*Hepatozoon canis*		Fox	Spain	AY150067	[1]
		*Dusicyon thous*	Brazil	AY461375	
*Hepatozoon felis*		Asiatic lion	India	ON075470	[50]
		Domestic cat	Uruguay	MT210597	[51]
		Domestic cat	Uruguay	MT210598	
*Hepatozoon martis*		*Martes martes*	Bosnia and Herzegovina	MG136687	[52]
		*Martes foina*	Croatia	MG136688	
*Hepatozoon pyramidumi*		*Echis pyramidum*	Saudi Arabia	MT025290	[12]
*Hepatozoon* sp.	Hap_1	*G. cheesmani*	Saudi Arabia	PV342392	Present study
	Hap_2	*G. nanus*		PV342388	Present study
	Hap_3			PV342393	
	Hap_4	*R. rattus*		PV342391	Present study
	Hap_5			PV342394	
	Hap_6	*A. niloticus*		PV342389	Present study
	Hap_7			PV342390	

## Data Availability

Sequence data have been deposited in GenBank under the accession numbers PV342388-PV342395.
